# Giant Controllable Magnetization Changes Induced by Structural Phase Transitions in a Metamagnetic Artificial Multiferroic

**DOI:** 10.1038/srep22708

**Published:** 2016-03-04

**Authors:** S. P. Bennett, A. T. Wong, A. Glavic, A. Herklotz, C. Urban, I. Valmianski, M. D. Biegalski, H. M. Christen, T. Z. Ward, V. Lauter

**Affiliations:** 1 Quantum Condensed Matter Division, Neutron Sciences Directorate, Oak Ridge National Laboratory, Oak Ridge, TN 37830, USA; 2 Materials Science and Technology Division, Oak Ridge National Laboratory, Oak Ridge, TN 37830, USA; 3 Laboratory for Neutron Scattering and Imaging, Paul Scherrer Institut, 5232 Villigen, Switzerland; 4 Department of Materials Science and Engineering, The University of Tennessee, Knoxville, TN 37996; 5 Center for Nanoscale Materials Science, Oak Ridge National Laboratory, Oak Ridge, TN 37830, USA; 6 Department of Physics, The University of California, San Diego, San Diego, CA 92110, USA

## Abstract

The realization of a controllable metamagnetic transition from AFM to FM ordering would open the door to a plethora of new spintronics based devices that, rather than reorienting spins in a ferromagnet, harness direct control of a materials intrinsic magnetic ordering. In this study FeRh films with drastically reduced transition temperatures and a large magneto-thermal hysteresis were produced for magnetocaloric and spintronics applications. Remarkably, giant controllable magnetization changes (measured to be as high has ~25%) are realized by manipulating the strain transfer from the external lattice when subjected to two structural phase transitions of BaTiO_3_ (001) single crystal substrate. These magnetization changes are the largest seen to date to be controllably induced in the FeRh system. Using polarized neutron reflectometry we reveal how just a slight in plane surface strain change at ~290C results in a massive magnetic transformation in the bottom half of the film clearly demonstrating a strong lattice-spin coupling in FeRh. By means of these substrate induced strain changes we show a way to reproducibly explore the effects of temperature and strain on the relative stabilities of the FM and AFM phases in multi-domain metamagnetic systems. This study also demonstrates for the first time the depth dependent nature of a controllable magnetic order using strain in an artificial multiferroic heterostructure.

While most focused research into the electric field control of magnetization has been centered on multiferroic oxide heterostructures, there is a rapidly growing interest into the radically different system of metallic FeRh for use in intelligently designed artificial multiferroics. Metamagnetic FeRh has been under scrutiny for such an application for it’s close to room temperature phase transition from AFM to FM ordering^1^. Though a material with a magnetic ordering change close to room temperature is not unique, the transition in FeRh thin films has some quite unusual characteristics. Differing to other systems, FeRh’s low temperature phase is antiferromagnetic (AFM) while its higher temperature state is of ferromagnetic (FM) ordering^2^,^3^,^4^,^5^,^6^. FeRh’s AFM→FM transition is also distinctive for its intimate connection to a coinciding ~1.0% volumetric expansion^7^,^8^,^9^, and a clear change in electrical magneto resistivity^10^,^11^, leading to the recent interest in its use in possible antiferromagnetic electronics^11^,^12^. Even more interesting is the recently reported control of magnetic ordering temperature using a small piezo-electric substrate induced strain^13^,^14^,^15^. The nature of the phase transition in these heterostructures has been studied by methods such as x-ray magnetic circular/linear dichroism (XMLD/XMCD)^16^,^17^, x-ray photoemission electron microscopy (XPEEM)^15^,^18^ and electrical transport^10^,^19^,^20^, however these methods are insensitive to the fine details of strain effects which should be strongest at the buried interfaces and may vary through the thickness of the film. Mapping of the depth dependent density and magnetization under different temperature and strain configurations is crucial to understanding the metamagnetic effects created by an isothermal strain change.

In this study samples were grown with the goal of inducing and stabilizing stresses in the system in order to maximize the films response to an external strain induced stimulus. For this, a post anneal rapid thermal quench was implemented using a laser substrate heater after sputter deposition (see methods section for growth details). This produces a giant magnetization response (measured to be as high has ~25%) when subjected to the interfacial strain changes induced at two structural phase transitions of the BTO 001 single crystal substrate (orthorhombic (O) → rhombohedral (R) and orthorhombic → tetragonal (T)). These magnetization changes are the largest seen to date to be controllably induced in the FeRh system^4^,^20^,^21^,^22^,^23^. Furthermore this shows, for the first time, the depth dependent nature of a controllable magnetic order using strain.

Here we coerce a unique structure and properties into the BTO-FeRh system by varying the annealing conditions. A first group of reference films was grown using the common growth methods^2^,^7^,^8^ by magnetron sputter from a stoichiometric FeRh target. These films show a relatively narrow AFM→FM transition region that spans about 20 K from 320 K→340 K. Figure 1(a) depicts the thermomagnetic characteristics of the film obtained by superconducting quantum interference device (SQUID) magnetometry. While this transition temperature region is lower than that for bulk FeRh, where it occurs at ~450 K^24^,^25^, such a decrease is common to FeRh thin films and has been said to depend highly on the substrate used and the initial strain state of the film^9^,^14^,^10^. Of interest to this work are the features at ~190 K and ~285 K shown as insets of Fig. 1(a). These changes in magnetization occur at the structural phase transitions of the underlying BTO (001) single crystal substrate. Specifically, the magnetization response occurring at ~190 K corresponds to a transition between the low temperature rhombohedral crystal and the orthorhombic crystal phase. At the higher temperature of ~285 K there is another change in the magnetization at the transition from the orthorhombic phase to the room temperature tetragonal phase. These responses are significant because they correspond to a change in the intrinsic magnetic ordering of the AFM state, attributed completely to strain changes induced by the structural transitions of the substrate.

To maximize the films response to external strain induced stimulus an *in-situ* rapid thermal quench was implemented after a 730 °C post growth anneal. In stark contrast to the sample in Fig. 1(a) are the thermomagnetic measurements of a film subject to this rapid thermal quench shown in Fig. 1(b). They demonstrate an increase in the overall magnetization maximum, from ~550 emu/cc at 380 K for the unquenched sample, to ~1020 emu/cc at 300 K for the quenched sample. Additionally, the transition temperature is found to be greatly reduced to approximately ~180 K, and the transition region now spans more than 200 K (from ~180 K to at least our maximum measured temperature of 380 K). Within this transition region the AFM and FM phases are largely metastable, and by shifting the transition to lower temperature and increasing the regions span, the region of instability now covers the structural transitions of the BTO substrate. Remarkably this has resulted in the amplitude of the magnetic response to increase more than 100 fold to be close to 25% of the highest measured saturation magnetization of the sample. The magnetization change occurs in both the forward and reverse temperature sweep directions and their amplitude depends strongly on both the applied H-field and temperature based sample measurement history. Figure 1(b) (red line) also shows how applying a saturation magnetic field of 1T, then cooling the sample in zero field (ZFC) before measurement, helps to increase the magnitude of the magnetization response at some of the crystal transitions. There is yet to be determined a strong explanation for this effect, however evidence hints toward a temperature/phase dependent concentration variation of ferroelectric domain orientations in the underlying BTO being a contributor.

In order to establish a comprehensive picture and to fully understand the nature of this strain induced magnetization response it is necessary to probe the magnetization of the film as a function of distance from the substrate. To do this polarized neutron reflectometry (PNR) was used, which provides the unique ability to measure simultaneously both the magnetization depth profile and (due to a high neutron scattering contrast between Fe and Rh) also the element density depth profile^26^. Neutron measurements were performed at temperatures both above and below the two phase transitions of interest after a zero field cool (ZFC) at 175 K, 200 K, 280 K, 295 K (marked in Fig. 1(b) green); and at 200 K after a field cool (FC) in 5 kOe (marked in Fig. 1(b) with black). The PNR experiments were performed in a saturation magnetic field of 5 kOe oriented parallel to the sample to clearly trace the two, FM and AFM, phases. Figure 2(a) shows the PNR data for two neutron spin polarizations parallel (+) and anti-parallel (−) to the external magnetic field along with fits to the data. The depth profiles of the combined elemental density distribution and in-plane magnetization distributions directly correspond to the depth profiles of the nuclear and magnetic scattering length densities, respectively (Fig. 2(b–e)).

The depth profiles at each temperature show some distinct features and similarities. PNR reveals that the film contains two main parts with drastically different properties. The top part of the film shows an increased magnetization toward the surface and is persistently ferromagnetic even at the lowest temperature of 175 K where stoichiometric FeRh would be in the AFM phase. This region of the film has a higher scattering length density (shown in Fig. 2(c)), which is a signature of an Fe – rich region (whose NSLD of 8.01 × 10^−6^ Å^−2^ is much higher than Rh’s at 4.27 × 10^−6^ Å^−2^). Another feature of the magnetization profiles common to all temperatures is a persistent interfacial FM layer of ~10 nm at the substrate interface. Such an interfacial magnetization has been seen before in FeRh^27^,^28^,^29^, and recently confirmed in Pd doped FeRh films^30^.

The key experimental observation is that, although the two magnetization changes shown in the SQUID measurements in Fig. 2(d,e) are comparable in overall amplitude, the PNR results unveil precisely where these moment changes occur in the film, and that they have different origins for the two transitions. In cooling through BTO’s O→R transition (200 K→175 K) the FeRh consequently undergoes a large instantaneous drop in magnetization, with the largest drop being after a ZFC shown in Fig. 1(b))^24^. This is shown in Fig. 2(d) to occur uniformly through the whole thickness of the film, accompanied by only a small increase in magnetization of the persistently ferromagnetic interfacial region. In contrast, when the film is heated through the O→T phase transition (295 K→280 K), there is a large increase in the magnetization concentrated in the bottom half of the film shown in Fig. 2(d). In analyzing room temperature XRD data in Fig. 3(b) we can determine that the majority of the BTO substrate contains ferroelectric a-domains based on the higher relative intensity of the BTO 300/030 peak when compared to the BTO 003 peak (see Fig. 3(b) inset). We also know that on entering the O phase there are 3 possible domain orientations, (with two of them being equivalent if indexed as a primitive unit cell with a = c > b & β ≠ 90°)^31^. This means that, in heating from the orthorhombic phase there is an in-plane surface expansion on the order of ~0.075% and ~0.75% across the O→T transition (see Fig. 3(c)). This small expansion results in a substantial increase in the overall magnetization (shown in both SQUID and PNR data points in Fig. 1(b)). In particular PNR shows that this change happens almost solely in the bottom half of the film; effectively inducing a massive transformation of most of the remaining AFM phase to FM ordering (Fig. 2(e)). This is in direct agreement with reports that tensile strain of FeRh induced by growth on different lattice mismatch engineered substrates stabilizes the FM phase by lowering the transition temperature^32^,^33^. The measurements we present here of magnetizations response to acute isothermal strain changes performed at different temperatures expand on these observations in showing a strong coupling between both strain and temperature parameters on the stability of the magnetic ordering in FeRh.

It’s also interesting to note that this large portion of sample changing to the FM phase at the O→T transition is also accompanied with a reduction in the scattering length density, which is shown clearly in the nuclear scattering length density (NSLD) profiles as the red curve in Fig. 2(c). Due to the unknown contribution of local density variation between AFM and FM domains these NSLD profiles cannot be directly correlated to a precise depth profile of chemical composition. Nevertheless we can assume that the chemical composition remains constant in the temperature range measured here. Meaning that this effect is a direct result of the ~1% lattice expansion associated with the AFM-FM transition in FeRh, and (in a smaller amount) by the small tensile strain change from the substrate.

There is also a large magnetization change at the O-R phase transition, where, depending on the substrate’s domain structure, either tension or compression would be acting onto the film. Interestingly, our PNR data shows that the changes occur predominantly within the FM portion (top layer) of the film, and can thus not be interpreted in the same framework as the effects at the T-O transition. SQUID measurements alone would not have been able to differentiate between the two, showing the importance of using PNR to obtain magnetic depth profiles, especially in systems like the present one where minute deviations from stoichiometry and vertical stoichiometry gradients can fundamentally alter the material’s properties.

In conclusion we’ve uncovered that the magnetic state in FeRh undergoes large controllable magnetization changes when grown on BTO 001 substrates. This effect has been demonstrated as the system is cooled/heated through the O→R and O→T crystalline transitions of BTO, inducing a strain on the intimately clamped FeRh film. PNR has revealed that a massive transformation occurs in the stoichiometric bottom half of the film close to room temperature (~285 K) at the O→T transition. Analysis has revealed that the magnetostructural and temperature dependent nature of FeRh is responsible for this effect; unveiling a competition between the two parameters of temperature and strain. The present findings provide a new discovery of a strong strain mediated handle for the control of magnetic ordering in FeRh films, and suggests exploring similar coherent material systems that can be implemented in thin film form.

## Methods

### Sample Growth

**Figure 3:**
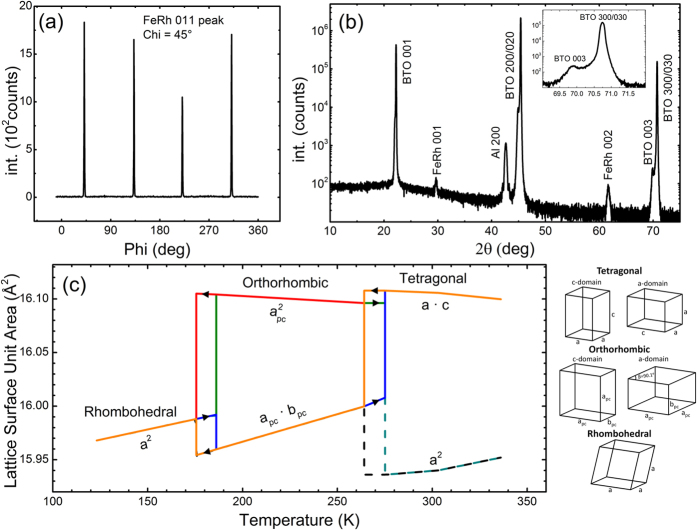
(**a**) XRD in-plane pole figure of the (011)_pc_ FeRh peak taken at a chi angle of 45° from rapidly quenched sample. (**b**) High angle out of plane XRD scan of the FeRh/BTO heterostructure in the rapidly quenched sample from 2θ = 10°–80° with peak index labels showing the highly ordered single crystalline nature of the FeRh film. Indexed Al 200 peak is from the XRD sample holder. Inset shows peak intensity difference between BTO 003 and BTO 300/030 peaks indicating a-domain structure in the tetragonal. (**c**) Plot showing the in-plane unit cell surface area of BTO for different domain orientations as a function of temperature (data calculated from unit cell parameters delineated by H.F. Kay & P. Vousden^23^). Dashed lines show c-domain surface areas for BTO not present in these samples as indicated in Fig. 3(b).

All samples were grown by magnetron sputter from a 50:50 composition FeRh sintered target on barium titanate BTO single crystal 001 oriented substrates in a chamber equipped with a laser substrate heater. The film growth process was performed in an argon atmosphere of ~5 mTorr and a growth temperature of 630 °C, followed by a post-growth 730 °C *in-situ* anneal for 1 hr in high vacuum. For the sample exposed to a rapid thermal quench the laser substrate heater was turned off after ~5 min at 730 °C, rapidly cooling the substrate to room temperature at >200  °C/min through the cubic → tetragonal transition of the BTO 00 substrate. The crystal structure and quality of the films was also characterized by X-ray diffraction (XRD). Pole figure measurements (Fig. 3(a)) about the in-plane (011)_pc_ peak (where “pc” stands for pseudocubic notation) and out of plane scans (Fig. 3(b)) indicate good crystallinity and an epitaxial relationship with the BTO substrate.

### Polarized Neutron Reflectometry (PNR) Experiments

PNR measurements were performed on the Magnetism Reflectometer at the Spallation Neutron Source (SNS) at Oak Ridge National Laboratory. This is a time of flight instrument with a wavelength band of λ ~ 2–8 Å of highly 98.5% polarized neutrons. The data was recorded with the position sensitive detector and the reflected and scattered intensity was normalized to the intensity spectrum of the incident beam. The reflected and scattered neutron data is collected in a two-dimensional map as a function of *p*_i_ and *p*_f_ where *p*_i_ = 2 π sinα_i_/λ and *p*_f_ = 2 π sinα_j_/λ are the perpendicular components of the neutron wave vectors, with α_i_/α_f_ being the incident/scattered angles and λ is neutron wavelength. The specular reflectivity is extracted from the two-dimensional intensity map as a function of incident momentum transfer normal to the sample surface, Q_z_ = *p*_i_ + *p*_j_ = 4 π sinα_i_/λ. During the experiment an external magnetic field of 0.5 T is applied in-plane of the film to saturate the sample parallel to the neutron polarization direction. A closed cycle refrigerator Displex^TM^ Model DE-204 from Advanced Research Systems with the SNS custom designed hot stage was used to perform experiments in the temperature range of 5 K–750 K.

The experiential data is used to extract the neutron scattering length density (NSLD) values. The depth dependence of the SLD profile is obtained by fitting the reflectivity data. For the spin-polarized (μ+ for spin-up and μ- for spin-down) neutron beam the reflectivity data for the two spin states are recorded as functions of momentum transfer Q_z_. In this case, the propagation of the neutron beam can be presented by optical formalism^34^, in which the interaction between the radiation and the medium is described by the Fermi pseudo potential^35^,^36^


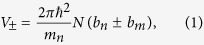


where ± denotes the spins of neutrons parallel and anti-parallel to the external field H, respectively; N represents number density of the material (~Å^−3^), m_n_ neutron mass, b_n_ and b_m_ denote the nuclei and magnetic scattering amplitude (~Å), respectively. From the fits to the two reflectivity curves (R^+^ and R^−^), the depth-dependent nuclear (Nb_n_) and magnetic (Nb_m_) scattering length density profiles (NSLD and MSLD), respectively, are determined. In particular, when measuring at magnetically saturated state, the MSLD (Nbm) obtained is linked to M_s_^37^ and allows its direct evaluation*.

The reflectivity data was fit with Python based software GenX^38^. Fitting of the reflectivity data was first attempted with the minimum number of parameters (substrate, film and surface layer). Each layer was described with 3 parameters (NSLD, MSLD and thickness). The model was then refined by successively adding sublayers to fit the details of the magnetic and nuclear structure until a good statistical fit to the data was achieved (12 layers total). The fit of R^+^ and R^−^ was performed simultaneously for each temperature dataset. To strengthen further the quality of the fit, we performed simultaneous fitting of the 200 K(after FC) and 280 K datasets, which are both in the orthorhombic crystal phase of the BTO substrate. To verify the true solution, parameter values were varied within physical uncertainties and compared to the experimental data within the error bars to estimate the maximum possible deviation of the obtained parameters. The estimated error is not exceeding 5% off the obtained parameter values.

*The magnetic scattering length density MSLD (or Nb_m_ in Eq (1)) is the same physical parameter as the magnetization (M), via the following conversion in the cgs unit system, 3.5 × 10^8^ × Nb_m_ (Å^−2^) = M (emu/cm^3^).

## Additional Information

**How to cite this article**: Bennett, S. P. *et al*. Giant Controllable Magnetization Changes Induced by Structural Phase Transitions in a Metamagnetic Artificial Multiferroic. *Sci. Rep.* **6**, 22708; doi: 10.1038/srep22708 (2016).

## Figures and Tables

**Figure 1:**
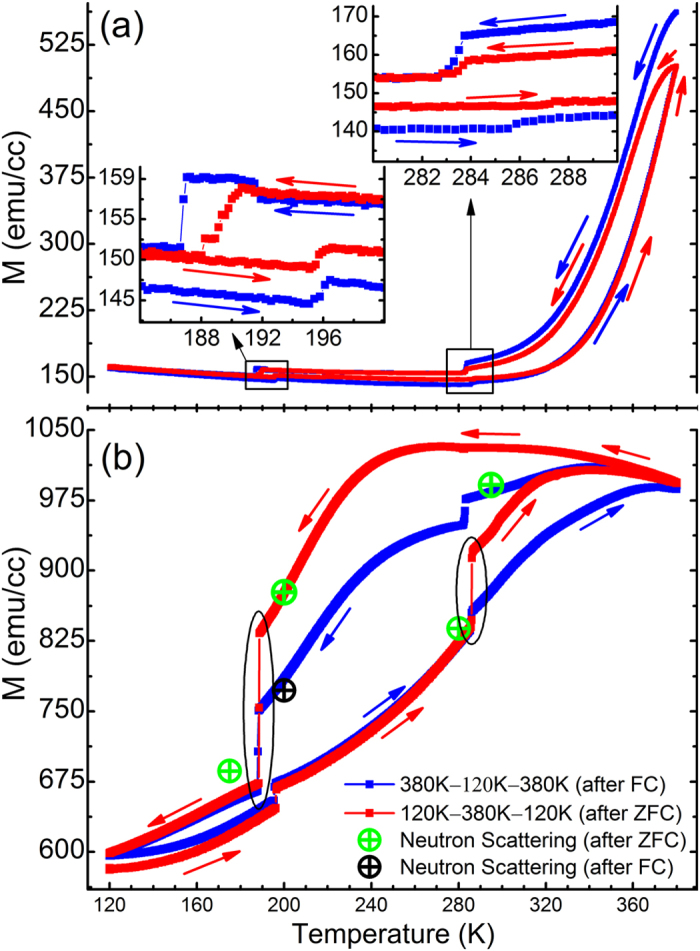
(**a**) SQUID thermomagnetic measurements of film grown by common methods for both field cooled (FC) and zero field cooled (ZFC) measurements. For the ZFC measurements the sample was initially saturated at room temperature in a 1 T magnetic field. Then, after cooling in zero field, measurements were taken with 5 kOe applied field (shown in red). The FC sweep was taken directly following the ZFC and was also measured in 5 kOe applied field (shown in blue). The inset is an expanded view of the magnetization changes detected at the crystalline transitions of the underlying BTO substrate. (**b**) SQUID thermomagnetic measurements of a rapidly quenched sample obtained in an identical procedure as that used for the film shown in Fig. 1(a). Ovals indicate the substrate strain configurations that resulted in the largest magnetization change. Data shown by green and black symbols correspond to the overall sample magnetization obtained from the polarized neutron reflectivity data by integrating the magnetization profiles shown in Fig. 2(c–e).

**Figure 2:**
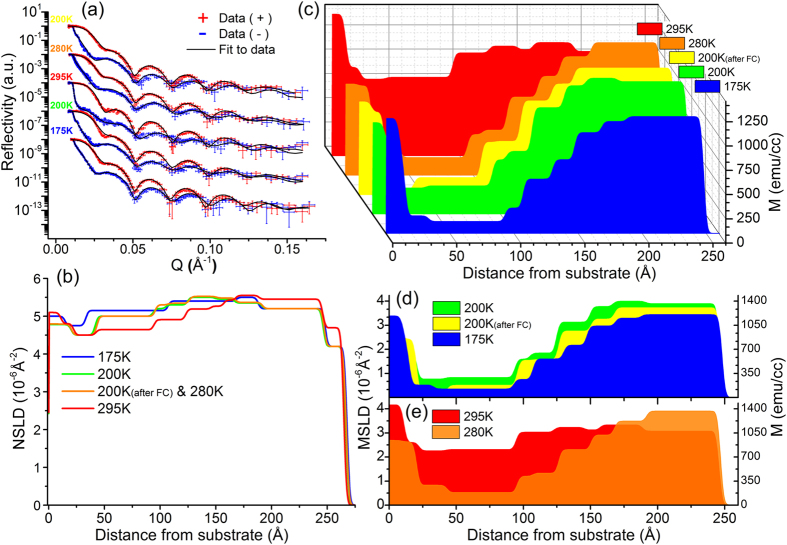
Polarized Neutron Reflectivity (PNR) data and fit results for the same rapidly quenched FeRh/BTO sample as measured in Fig. 1(b). All data was collected after a ZFC for each temperature unless otherwise indicated (i.e. FC) (**a**) PNR curves as the function of the momentum transfer. The order of the data reflects the sequence by which it was measured following the temperature sweep directions shown in Fig. 1(b), (starting with 200 K after FC and ending at 175 K). The experimental data are shown as intersecting error bars. The fitting results are presented with the solid lines. All structural and magnetization parameters were fit individually for each temperature (accept for 200 K and 280 K whose structural parameters were fit simultaneously). (**b**) Nuclear scattering length density (NSLD) depth profiles obtained from the data fits. (**c**–**e**) show the depth profiles of the magnetization (right y axis) and the magnetic scattering length density (MSLD) (left y axis) obtained from the corresponding fit shown in Fig. 2(a).
